# Functionalized zein nanoparticles targeting neonatal Fc receptor to enhance lung absorption of peptides

**DOI:** 10.1007/s13346-022-01286-4

**Published:** 2023-01-01

**Authors:** Fatima Hameedat, Soraia Pinto, Joana Marques, Sofia Dias, Bruno Sarmento

**Affiliations:** 1 i3S – Instituto de Investigação e Inovação em Saúde, University of Porto, Rua Alfredo Allen 208, 4200-135 Porto, Portugal; 2 NANOMED EMJMD, Pharmacy School, Faculty of Health, University of Angers, Angers, France; 3 INEB – Instituto de Engenharia Biomédica, University of Porto, Rua Alfredo Allen 208, 4200-135 Porto, Portugal; 4 ICBAS – Instituto de Ciências Biomédicas Abel Salazar, University of Porto, Rua Jorge Viterbo Ferreira 228, 4050-313 Porto, Portugal; 5 FFUP – Faculty of Pharmacy, University of Porto, Rua de Jorge Viterbo Ferreira 228, 4050-313 Porto, Portugal; 6 IUCS – CESPU, Rua Central de Gandra 1317, 4585-116 Gandra, Portugal

**Keywords:** Peptides delivery, Neonatal Fc receptor, Lung absorption, Targeted nanoparticles, Zein nanoparticles, PEGylation

## Abstract

Peptides have a distinguished therapeutic potential for several chronic conditions, and more than 80 peptides exist in the global market. However, most of these marketed peptide drugs are currently delivered intravenously or subcutaneously due to their fast degradation and limited absorption through non-invasive routes. The pulmonary route is favored as a non-invasive route. Neonatal Fc receptor (FcRn) is expressed in adult human lungs and has a role in enhancing the pulmonary absorption of monoclonal antibodies. In this work, we developed and characterized candidate protein delivery systems for the pulmonary administration of peptides. The prepared bare and loaded zein nanoparticles (ZNPs), targeted, physically, and covalently PEGylated ZNPs showed hydrodynamic diameters between 137 and 155 nm and a narrow distribution index. Insulin, which was used as a protein model, showed an association efficiency of 72%, while the FcRn-targeted peptide conjugation efficiency was approximately 68%. The physically adsorbed poloxamer 407 on insulin-loaded ZNPs showed slower and controlled insulin release. The in vitro cell culture model consists of the NCI-H441 epithelial cell line, which confirmed its expression of the targeted receptor, FcRn. The safety of ZNPs was verified after incubation with both cell lines of the in vitro pulmonary model, namely NCI-H441 and HPMEC-ST1.6R, for 24 h. It was observed that targeted ZNPs enhanced insulin permeability by showing a higher apparent permeation coefficient than non-targeted ZNPs. Overall, both targeted PEGylated ZNPs showed to be suitable peptide carriers and adequately fit the demands of delivery systems designed for pulmonary administration.

**Graphical Abstract:**

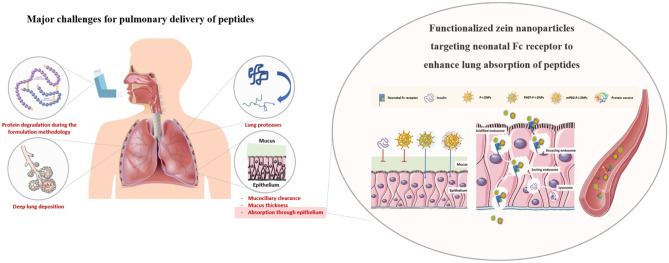

**Supplementary Information:**

The online version contains supplementary material available at 10.1007/s13346-022-01286-4.

## Introduction

Proteins and peptides have a great therapeutic potential for several chronic conditions such as diabetes, cancer, rheumatoid arthritis, and leukemia; therefore, their delivery has attracted a great deal of interest. Hitherto, more than 150 peptides reached clinical development, besides more than 80 peptide drugs reached the global market [1]. However, most of the marketed peptides are currently delivered intravenously or subcutaneously due to the fast degradation and limited absorption in the gastrointestinal and respiratory tracts [1]. In this sense, non-invasive routes are preferred for drug delivery, specifically the pulmonary route. For instance, the drug delivery systems designed for it were able to deliver high peptide concentrations for the local treatment of respiratory disorders with low systemic side effects and a faster clinical response [2]. In the case of systemic delivery, a similar therapeutic effect could be achieved compared to the intravenously administered dose and higher than other non-invasive routes since drugs are absorbed more efficiently through the lung [3]. This might be a consequence of their ability to bypass the classic oral delivery barriers that affect their absorption, such as dietary complications, interpatient metabolic differences, harsh conditions of the gastrointestinal tract, intestinal mucus, and epithelial barriers [2]. Therefore, this work proposes to take advantage of these properties of pulmonary delivery to enhance the absorption of peptides. However, despite the potential benefits of their delivery to the lungs, several challenges can compromise an optimum delivery [2]. On the production scale, it is challenging to produce inhalable protein therapeutics, which may undergo various degradation mechanisms during manufacturing processing and/or storage; hence, the formulation methodology should avoid harsh production conditions. Biologically, the inhaled proteins would be subjected to clearance by lung proteases that can degrade them before reaching the target site in the lung. In addition, the mucociliary clearance could eject them out of the lungs, by the coordinated beating of cilia lining the trachea and bronchi [4]. In certain lung pathologies, the mucus thickness increases, such as from 2 to 30 µm in normal cases to more than 260 µm in cystic fibrosis, which hinders the inhaled protein from reaching the site of action [2]. Notably, inhaled proteins have a relatively poor absorption through the epithelial layer to reach the deep parenchyma lung. Another crucial challenge is the lung deposition pattern, which is influenced by the device design at the production scale, namely the characteristics of the inhalable drug, particle size, and physicochemical properties of the formulation, and also biologically influenced by the specific disease state, which might affect the patient breathing patterns, and lung geometry, such as airway diameter and the number of alveoli [5]. Owing to these challenges, if inhaled proteins are not able to withstand the lung’s clearance mechanisms, it would be worthless to have a good lung deposition pattern. The conventional pulmonary drug delivery systems are limited due to these obstacles; therefore, the need to develop a targeted delivery platform is required. In this study, the peptide model used was insulin. Although the FDA approved two inhalable formulations for insulin, namely Exubera^®^ (withdrawn) and Afrezza^®^, both are not intended to meet basal insulin (intermediate or long-acting form) requirements [6], and the bioavailability of Afrezza^®^ is 20–30% [7].

The neonatal Fc receptor (FcRn) is a major histocompatibility complex class I (MHC-I)-like molecule that primarily functions as a dual-binding receptor of immunoglobulin G (IgG) and albumin to protect them from catabolism and mediates the transport of IgG across epithelial cells. FcRn is known as a player in the transfer of passive immunity from mother to fetus and neonate, which is involved in several biological and immunological processes. Additionally, FcRn is expressed in adult human in different sections of the intestine, including the colon and ileum [8]. Moreover, it has been detected in the lungs of several species, specifically, in the bronchial epithelial cells of adult humans, non-human primates, rats, mice, and cows [9, 10]. It has been described that its ability to enhance the pulmonary absorption of Fc-fusion is via FcRn-mediated transcytosis in the upper and central respiratory tract [11, 12]. In addition, it plays a key role in the recycling of monoclonal antibodies (mAbs) in the airways. Guilleminault et al. showed that using the lung tissue of FcRn-wild-type, the mean residence time of cetuximab was longer than that in FcRn-knockout animals by 10 times, providing evidence that FcRn plays a key role in mAb distribution and pharmacokinetic, and had a greater contribution to its recycling than to the transcytosis in the airways [13]. Regardless of the mechanism involved, the ideal location for the absorption of mAbs could be the upper and central airways, where FcRn is expressed [12]. Interestingly, its unique mechanism to prolong the half-life of IgG and albumin has paved the way to engineer novel therapeutics [9, 12, 14]. In order to target the FcRn, the candidate nanocarrier was decorated with a specific FcRn-targeted ligand. The selection of the ligand follows specific criteria that augment its binding affinity at acidic pH and avoid the increase in receptor affinity at neutral pH [14]. Furthermore, although FcRn expression in human lung tissues and its role were defined for decades, a quantitative comparison between different lung cell lines in terms of its expression is lacking in the literature. Hence, the choice of the ideal cell line model needs to be studied.

The delivery of peptides using a carrier decorated with peptides is challenging because they might be degraded once exposed to certain external stresses, such as high temperature, extreme pH, organic solvent, and salt [15]. Therefore, to obtain a targeted delivery system, the nanocarrier of choice should be suitable to encapsulate peptides with a liable method and decorate its surface with ligands. The naturally amphiphilic protein zein was selected to encapsulate the peptide of interest to investigate its potential to be used as a nanocarrier for proteins and peptides. It is isolated from corn seeds and has biodegradability and biocompatibility characteristics. In fact, it is a Generally Recognized As Safe (GRAS) protein by FDA and has potential use as an excipient in food production, as well as other pharmaceutical applications discussed in the literature [16, 17]. On top of that, it is cultivated, easily available, and economically friendly. As a nanocarrier, it has attracted the attention of researchers to be used for both hydrophilic and lipophilic drugs [18–21]. Plenty of methodologies were described in the literature, such as the antisolvent precipitation method, antisolvent co-precipitation method, layer-by-layer method, solvent evaporation method, pH-driven method, and desolvation method “liquid–liquid dispersion method.” The choice of the production methodology depends on many factors but mainly the characteristics of the drug of interest to encapsulate [16]. In this study, the desolvation method will be used to encapsulate the peptide model, insulin, which was previously performed by Reboredo et al. [18]. The authors showed high association efficiency and improved hypoglycemic effect with an oral bioavailability of 10% for the physically PEGylated zein nanoparticles (ZNPs) in diabetic rats [18]. Despite the advantages of using zein as a nanocarrier, it has mucoadhesive properties, limiting its ability to cross the mucus barrier of the lung. Perhaps, this limitation hindered the researchers to use it as a nanocarrier for pulmonary delivery; therefore, designing a mucopenetration ZNPs is required. Therefore, our goal was to produce PEGylated ZNPs loaded with insulin and functionalized with an FcRn-targeted ligand to increase their penetration through the mucus and enhance peptide bioavailability. Particularly, modifying the nanoparticles (NPs) surface with polyethylene glycol (PEG), physically using poloxamer 407 and chemically using methoxy PEG (mPEG), with specific length and density adopted from the literature, was investigated. Nevertheless, the used coating strategy is the ligand buried under the PEGylated layer which is one of the stealth active targeting NPs strategies.

In this study, we evaluated the PEGylation impact on the apparent permeability of targeted insulin-loaded ZNPs. Therefore, both systems with covalent and adsorbed PEGylated-targeted ZNPs were produced and characterized. Furthermore, an in vitro pulmonary model, developed by Costa et al. [22], was used to investigate the permeability of insulin loaded to both PEGylated nanosystems and determine what is the most suitable nanosystem for the delivery of insulin via the pulmonary route.

## Materials and methods

### Materials

Ethanol (absolute degree) and hydrochloric acid (HCl) are common reagents supply. MES buffer was purchased from ACROS Organics (New Jersey, NY, USA) and poloxamer 407 (Kolliphor^®^ P407) from BASF (Geismar, LA, USA). An FcRn-targeted peptide with the following amino acid sequence, CQRFVTGHFGGLYPANG, was requested from GenScript Biotech (Leiden, Netherlands). Zein, insulin, sodium phosphate dibasic heptahydrate, sodium phosphate monobasic monohydrate, N-(3-dimethylaminopropyl)-N′-ethylcarbodiimide hydrochloride (EDC·HCl), N-hydroxysuccinimide (NHS), O-methyl-O′-succinylpolyethylene glycol 5′000 (mPEG-COOH), gelatin from porcine skin, endothelial cell growth supplement (ECGS), dexamethasone (Dex), M-199 medium, and sulfuric acid (H_2_SO_4_) were all purchased from Sigma-Aldrich (St. Louis, USA). Paraformaldehyde (PFA) was purchased from Electron Microscopy Sciences (Hatfield, PA, USA). Heparin sodium salt from porcine intestinal mucosa was purchased from Alfa-Aesar (Haverhill, USA). Matrigel^®^ basement membrane matrix was purchased from Corning (New York, USA). Insulin-transferrin-selenium (ITS), Dulbecco’s Modified Eagle’s Medium (DMEM), Roswell Park Memorial Institute (RPMI)-1640 medium, and Hanks’ Balanced Salt solution (HBSS) were purchased from Gibco (Waltham, USA). Fetal bovine serum (FBS) was purchased from Biochrom AG (Berlin, Germany). Human FcRn antibody (MAB8639) was obtained from Bio-Techne (Minneapolis, USA), goat anti-mouse Alexa 594 (A-11020) from Invitrogen (California, USA), and anti-insulin + proinsulin mouse monoclonal capture antibody (ab8304) and HRP anti-insulin + proinsulin mouse monoclonal antibody (ab28063) from Abcam (Cambridge, UK).

### Nanoparticles preparation method

#### Preparation of zein nanoparticles

**Fig. 1:**
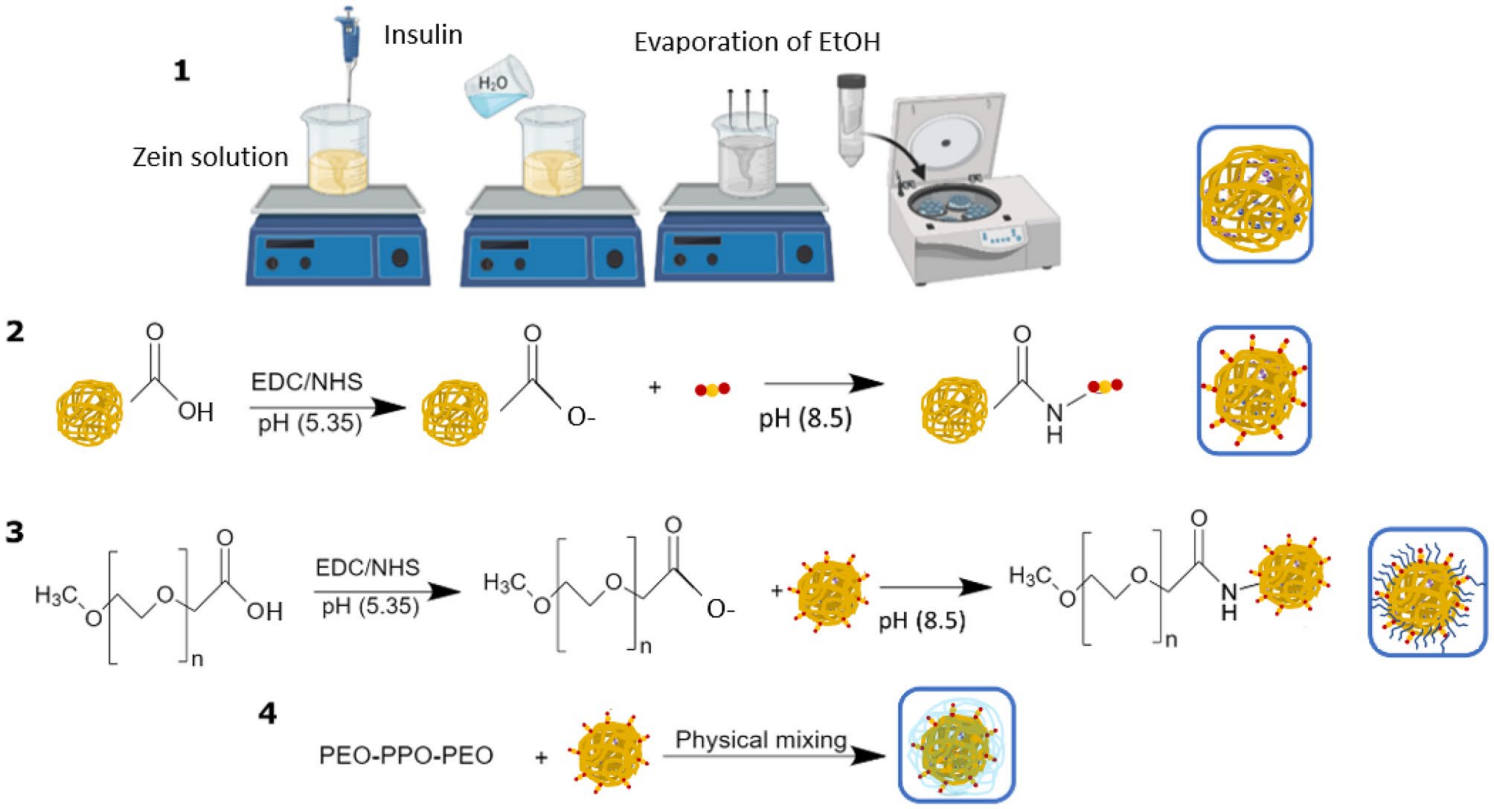
Methodology of preparation of (**1**) insulin-loaded ZNPs (i-ZNPs) using desolvation method; (**2**) FcRn-targeted i-ZNPs (F-i-ZNPs) using carbodiimide chemistry; (**3**) covalent PEGylated F-i-ZNPs (mPEG-F-i-ZNPs) using carbodiimide chemistry; and (**4**) physically adsorbed PEO-PPO-PEO (P 407) polymer F-i-ZNPs (P407-F-i-ZNPs). The figure was made using Adobe Illustrator and ChemDraw

A desolvation procedure previously described [18] was applied to prepare ZNPs with minor modifications (Fig. 1(1)). Briefly, 5 mL of a hydroalcoholic solution (61% (v/v) ethanol in water) was used to dissolve 50 mg of zein under continuous magnetic stirring, while 5 mg of insulin were dissolved in 0.5 mL of freshly prepared 10 mM HCl aqueous solution. Both solutions were mixed under continuous magnetic stirring for 30 min, and then the desolvation step of zein was induced by the addition of an equivalent volume of 5 mL of acidified Milli-Q^®^ ultrapure water at pH 5.3, under continuous magnetic stirring at 400 rpm. The evaporation of ethanol occurred under the fume hood for 4 h. Afterward, the suspension of ZNPs was collected and centrifuged at 2000 g for 30 min at room temperature (RT) through a filter device with a molecular weight cutoff of 100 kDa (Amicon Ultra filter, Ultracel membrane with 100,000 MWCO, Millipore Corporation, Bedford, USA). Empty ZNPs were prepared using the same protocol, but without insulin.

#### Selection of FcRn-targeted ligand

The FcRn-targeted ligand selected to functionalize the ZNPs was based on the SYN746 peptide framework (QRF**C**TGHFGGLYP**C**NGP), developed in Mezo et al.’s work [23]. This cyclic peptide was further converted to a linear peptide (QRF**V**TGHFGGL**Y**PANG) by replacing both cysteine residues with valine and alanine, at 4 and 14 positions, respectively, as previously described by Sockolosky et al. and Datta-Mannan et al. [24, 25]. A cysteine residue was added to the N-terminal of the linear FcRn-targeted peptide framework (**C**QRFVT**GHFGGLY**PANG) to facilitate its conjugation to the candidate nanocarrier by different possible chemistry reactions, namely maleimide-thiol and EDC/NHS chemistries.

#### Conjugation of FcRn-targeted ligand to zein nanoparticles

ZNPs were conjugated to the FcRn-targeted peptide mentioned in the previous section, the “Selection of FcRn-targeted ligand” section, using carbodiimide chemistry, in which the carboxyl (–COOH) groups of ZNPs were covalently linked to the primary amines (–NH_2_) groups of the peptide (Fig. 1(2)). Firstly, 1 mL of ZNPs (4 mg/mL) was diluted in acidic buffer (MES buffer, pH 5.3) and then reacted with EDC/NHS for 1 h to form ZNPs with amide bonds. The formed by-products were removed by filtration with Amicon tubes (100 kDa) at 2000 g for 15 min at RT. The formed ZNPs with amide bonds were added to the FcRn-targeted peptide (CQRFVTGHFGGLYPANG), previously dissolved in basic buffer (PBS, pH 8.5), under continuous stirring at RT for 1 h. The unconjugated FcRn-targeted peptide was removed with Amicon tubes (100 kDa) at 2000 g for 15 min at RT, and the functionalized ZNPs (F-ZNPs) were collected to a final volume of 1 mL and 4 mg/mL concentration.

#### Preparation of PEGylated functionalized zein nanoparticles

The production of PEGylated F-i-ZNPs was conducted using two methodologies: covalent binding of ZNPs with mPEG and physical mixture of ZNPs with poloxamer 407 (PEO-PPO-PEO (P 407)). Regarding the first methodology, mPEG-COOH:EDC:NHS in the molar ratio of 1:10:25 were dissolved in MES buffer (pH 5.35) to activate the -COOH group on mPEG-COOH by stirring for 3 h at RT (Fig. 1(3)). Then, the mixture was added dropwise to F-i-ZNPs for 1 h at RT to obtain covalently PEGylated targeted ZNPs (mPEG-F-i-ZNPs), whereas the physical adsorbance of P407 on F-ZNPs (P407-F-i-ZNPs) was obtained by adding dropwise 0.1 mL of F-i-ZNPs (20 mg/mL) into 0.9 mL of 0.1% (w/v) P407 and incubating overnight at 4 °C (Fig. 1(4)). In both formulations, samples were further filtered with Amicon tubes (100 kDa) at 2000 rpm for 15 min at RT to remove the free polymers. The formulations were stored at 4 °C until further use.

#### Freeze-drying of zein nanoparticles

The ZNPs of concentration 1 mg/mL and final volume 2 mL were poured into semi-stoppered glass vials with slotted rubber closures. ZNPs were initially frozen at −80 °C for 24 h followed by lyophilization using a Modulyo 4 K freeze-dryer (Edwards, Crawley, UK) at 0.09 mbar for 72 h. The temperature of the condenser surface was maintained at −60 °C ± 5 °C. After lyophilization, ZNPs were stored in a 4 °C chamber until future use.

### Physical–chemical characterization of zein nanoparticles

#### Hydrodynamic diameter, polydispersity index, and zeta potential

The freshly prepared ZNPs formulations were characterized regarding their hydrodynamic diameter and polydispersity index (PdI) through dynamic light scattering (DLS) and zeta potential (ZP) by laser Doppler electrophoresis (LDE), using the Zetasizer Nano ZS (Malvern Instruments, Malvern, UK). For that, samples were previously diluted in a ratio of 1:100 in 10 mM NaCl adjusted to pH 5 with HCl 0.5 M. All measurements were performed in triplicate at 25 °C. Data was collected and analyzed using Zetasizer software (version 7.12).

#### Stability of unloaded zein nanoparticles

The stability of the liquid suspensions of unloaded ZNPs stored in dark at 4 °C was evaluated until day 51 of production. The ZNPs were dispersed in 10 mM NaCl adjusted to pH 5 with 0.5 M HCl and characterized in terms of hydrodynamic diameter, PdI, and ZP, using Zetasizer equipment.

#### Morphological evaluation

The morphology of ZNPs, mPEG-F-ZNPs, and P407-F-ZNPs was confirmed by transmission electron microscopy (TEM). TEM images were obtained by placing 10 µL of 0.033 mg/mL NPs suspension of concentration on Formvar/carbon film-coated mesh nickel grids (Electron Microscopy Sciences, Hatfield, PA, USA) and left standing for 2 min. Then, the excess liquid was removed with filter paper. The visualization was carried out on a JEOL JEM 1400 TEM at 120 kV (Tokyo, Japan). Images were digitally recorded using a CCD digital camera Orious 1100 W (Tokyo, Japan), at the HEMS/i3S of the University of Porto.

The surface morphology of freeze-dried unloaded ZNPs was further studied by high-resolution scanning electron microscopy (SEM) with X-Ray Microanalysis and CryoSEM experimental facilities: JEOL JSM 6301F/Oxford INCA Energy 350/Gatan Alto 2500. ZNPs were mounted onto metal stubs with carbon tape and sputter-coated with a thin layer of gold/palladium using the SPI Module Sputter Coater equipment (Structure Probe, Inc., West Chester, PA, USA).

#### Attenuated total reflectance–Fourier transform infrared spectroscopy

In order to confirm the covalent bonds between FcRn-targeted peptide and ZNPs as well as mPEG-COOH and F-ZNPs, the Fourier transform infrared spectroscopy (FTIR) spectra of the raw materials and the NPs were obtained using a Fourier transform spectrophotometer IR Affinity-1S (Shimadzu, Kyoto, Japan) coupled to a Specac Golden Gate Attenuated Total Reflectance (ATR). For that purpose, NPs suspensions were placed over the diamond, and the reflectance spectra were obtained by scanning from 400 to 4000 cm^−1^ at 4 cm^−1^ of the resolution, and 32 scans per spectrum. The PerkinElmer Spectrum IR software was used to analyze the spectra.

#### Insulin association efficiency and drug loading

The association efficiency (AE) and drug loading (DL) of insulin in freshly prepared ZNPs were evaluated by an indirect method using ultracentrifugation. For that purpose, ZNPs were centrifuged at 30,000 g for 30 min at 4 °C, and the supernatants containing insulin were collected. The amount of loaded insulin into ZNPs was calculated by the difference between the initial amount of insulin used to produce the ZNPs and the remaining drug collected in the supernatant. An insulin calibration curve was prepared in a range of concentrations from 0.5 to 75 µg/mL, using the supernatant obtained from unloaded ZNPs as a solvent and as a blank, to remove possible interference from zein. Standards and samples were evaluated in triplicates by micro-BCA assay. The AE and DL were calculated by the following equations:

1 $$\mathrm{Association}\;\mathrm{fo}\;\mathrm{Efficiency}\;\left(\%\right)=\frac{\mathrm{Initial}\;\mathrm{mass}\;\mathrm{of}\;\mathrm{insulin}\!-\!\mathrm{Mass}\;\mathrm{of}\;\mathrm{recovered}\;\mathrm{insulin}}{\mathrm{Initial}\;\mathrm{mass}\;\mathrm{of}\;\mathrm{insulin}}\times100$$

2 $$\mathrm{Drug}\;\mathrm{loading}\left(\%\right)=\frac{\mathrm{Initial}\;\mathrm{mass}\;\mathrm{of}\;\mathrm{insulin}\!-\!\mathrm{Mass}\;\mathrm{of}\;\mathrm{recovered}\;\mathrm{insulin}}{\mathrm{Total}\;\mathrm{mass}\;\mathrm{of}\;\mathrm{the}\;\mathrm{ZNPs}\;\mathrm{and}\;\mathrm{insulin}}\times100$$

#### FcRn-targeted peptide conjugation efficiency

The conjugation efficiency (CE) of the FcRn-targeted peptide was determined by indirect method. Briefly, after unloaded ZNPs were functionalized with the FcRn-targeted peptide by carbodiimide chemistry, as described in the “Conjugation of FcRn-targeted ligand to zein nanoparticles” section, samples were transferred to Amicon tubes (100 kDa), and the unconjugated peptide was removed by centrifuging at 2000 g for 15 min at RT. Filtrates were collected, and the mass of conjugated peptide on ZNPs was determined by the difference between the initial mass of peptide and the mass of peptide detected in the filtrate. The amount of peptide non-conjugated was quantified using micro-BCA assay, with relevant controls, unconjugated ZNPs. Similarly to the insulin AE experiment, an FcRn peptide calibration curve was prepared in a range of concentrations from 0.5 to 50 µg/mL, using the filtrates obtained from unconjugated ZNPs as a solvent and as a blank, to remove possible interference from zein. Six independent experiments were performed (*n* = 6). The CE was determined by the following formula:

3 $$\mathrm{CE}\left(\%\right)=\frac{\mathrm{Initial}\;\mathrm{mass}\;\mathrm{of}\;\mathrm{peptide}-\mathrm{mass}\;\mathrm{of}\;\mathrm{peptide}\;\mathrm{in}\;\mathrm{the}\;\mathrm{supernant}}{\mathrm{Initial}\;\mathrm{mass}\;\mathrm{of}\;\mathrm{peptide}}\times100$$

#### In vitro release study

The release profile of insulin in i-ZNPs, F-i-ZNPs, mPEG-F-i-ZNPs, and P407-F-i-ZNPs was determined by incubating 2 mL of NPs (corresponding to an insulin concentration of 53 µg/mL) in phosphate buffer (20 mM) at pH 7.4. Aliquots of 400 µL were withdrawn from each formulation at specific time points (0.25, 0.5, 1, 2, 5, 8, and 24 h), and the same volume was replaced with warmed PBS. Samples were centrifuged at 13,000 g for 10 min at RT, and the supernatants were collected and quantified by micro-BCA assay to evaluate the amount of insulin released from each formulation throughout the experiment [26]. An insulin calibration curve was prepared in PBS, and samples were evaluated in triplicates with relevant controls (unloaded ZNPs). The experiment was performed at 37 °C, 120 rpm, and in sink conditions [27].

### Cell culture

#### Cell culture maintenance

Human lung carcinoma epithelial cell lines (A549, NCI-H441, and Calu-3 cells), human colorectal adenocarcinoma cell line (Caco-2 cells), and human cervical adenocarcinoma cell line (HeLa cells) were purchased from the American Type Culture Collection (ATCC, Manassas, USA). NCI-H441 cell line (passage number between 89 and 103) was cultured in T75 cm^2^ cell culture flasks with RPMI1640 culture medium supplemented with 10% (v/v) FBS, penicillin (100 IU/mL), streptomycin (100 mg/mL), 1% (v/v) non-essential amino acids (NEAA) 100 × concentrate (Gibco, Paisley, UK), and 1% (v/v) sodium pyruvate. Caco-2, HeLa, A549, and Calu-3 cell lines (passage number between 25 and 35, 20 and 35, 18 and 32, 83 and 93, respectively) were cultivated in T75 cm^2^ cell culture flasks with DMEM culture medium supplemented with 10% (v/v) FBS, penicillin (100 IU/mL), streptomycin (100 mg/mL), 1% (v/v) NEAA 100 × concentrate, and 1% (v/v) sodium pyruvate. HPMEC-ST1.6R cells were provided by professor C. James Kirkpatrick (Institute of Pathology, Johannes Gutenberg University of Mainz, Germany). The T75 cm^2^ cell culture flasks were coated with gelatin solution (0.2% (w/v)) for 20–30 min at 37 °C, and then HPMEC-ST1.6R cells (passage number between 34 and 48) were seeded. Cells were cultured with M-199 medium supplemented with 20% (v/v) FBS, penicillin (100 IU/mL), streptomycin (100 mg/mL), 1% (v/v) NEAA 100 × concentrate, 25 µg/mL of ECGS, and 25 µg/mL of heparin sodium salt. All cell lines were maintained in an incubator at 37 °C and 5% CO_2_ in a water-saturated atmosphere, and the medium was changed every 2–3 days. Once cells reached confluence (70–80%), they were washed with PBS and treated with trypsin–EDTA (for 5 min at 37 °C). After the inactivation of trypsin–EDTA by the addition of a complete medium, cells were centrifuged and seeded into new cell culture flasks.

#### Evaluation of FcRn expression by flow cytometry

FcRn expression on the membrane surface of NCI-H441, A549, and Calu-3 cells was evaluated through flow cytometry. The Caco-2 cell line was used as a positive control and the HeLa cell line as a negative control. Cells were grown inside an incubator with the humidified atmosphere at 37 °C and 5% CO_2_, and when reached 70–80% of confluence, cells were detached with Versene (Gibco Laboratories, Grand Island, NY), to maintain the integrity of cell surface molecules. Then, cells were counted and resuspended at 1 × 10^5^ cells/mL in FACS buffer (PBS supplemented with 10% FBS and 0.1% sodium azide), and 100 µL of cell suspension was added to each well of a round-bottom 96-well plate. The plate was centrifuged at 1500 rpm for 5 min at 4 °C. The supernatant was discarded, and the cells were resuspended in 50 µL of primary antibody (anti-mouse-FcRn (#MAB8639)) diluted in FACS buffer (2:1000 (v/v)), according to the manufacturer, for 2 h. Afterward, cells were washed in 100 µL of ice-cold FACS buffer and resuspended in a fluorescently labeled secondary antibody (goat anti-mouse Alexa 594 (#A-11020)), which was diluted in FACS buffer (2.5:1000 (v/v)) for 60 min at 4 °C. Samples were prepared in triplicates and analyzed using FACS Aria II (BD Bioscience, New Jersey, USA). Data were treated using FlowJo software (TreeStar Inc.) and presented as median fluorescence intensity (MFI).

#### Cell viability assay

NCI-H441 and HPMEC-ST1.6R cells were seeded in 96-well plates (*n* = 3) with a density of 4 × 10^4^ and 5 × 10^3^ cells per well, respectively [28, 29]. Cells were left to adhere and grow overnight in a 5% CO_2_ incubator at 37 °C. On the following day, the medium was discarded, and cells were incubated with different concentrations of ZNPs formulations diluted in the respective complete medium for 24 h at 37 °C and 5% CO_2_ incubator. A negative control, corresponding to cells incubated with 1% (v/v) Triton X-100 in medium, a positive control, consisting of cells incubated with medium, and a blank, comprising medium without cells, were also prepared. After 24 h of incubation, 10% (v/v) of Resazurin reagent diluted in the medium was added to each well and incubated at 37 °C for 2 h in an incubator with humidified atmosphere and 5% CO_2_. Resazurin is reduced to resorufin, resulting in a highly fluorescent compound, with an excitation wavelength of 530 nm and an emission wavelength of 590 nm. Fluorescence was measured using a microplate reader (*Synergy™ Mx HM550, BioTek*). Cell viability is expressed based on Eq. 4, in which the background signal refers to the blank.

4 $$\mathrm{Cell}\;\mathrm{viability}\left(\%\right)=\frac{\mathrm{Flourescene}\;\mathrm{signal}\;\mathrm{of}\;\mathrm{sample}-\mathrm{flourescence}\;\mathrm{signal}\;\mathrm{of}\;\mathrm{blank}}{\mathrm{Flourescence}\;\mathrm{signal}\;\mathrm{of}\;\mathrm{positive}\;\mathrm{control}-\mathrm{flourescence}\;\mathrm{ofblank}}\times100$$

#### Permeability assay

The permeability assay was performed using a lung monoculture model and a lung co-culture model, which were produced as previously described in the literature [22]. THP-1 macrophage-like cells were excluded from both models since the aim was to evaluate the permeability of ZNPs in a healthy model [22]. The monoculture model consists in culturing 1.0 × 10^5^ of NCI-H441 cell line on the apical side of the Transwell^®^ inserts of 12-well insert (cellQART^®^, Northeim, Germany) of 12 µm diameter, 1.1 cm^2^ area, and pore size of 1.0 µm. The co-culture model consists of NCI-H441 cell line cultured on the apical side (1.0 × 10^5^/insert), as well as HPMEC-ST1.6R cells seeded on the basolateral side (5.0 × 10^4^/insert) of a 12-well insert (cellQART^®^, Northeim, Germany) of 12 µm diameter, 1.1 cm^2^ area, and pore size of 1.0 µm. Before seeding HPMEC-ST1.6R cell line, the cell culture insert was previously coated with 50 µL/cm^2^ of Matrigel^®^ with a concentration of 4.0 mg/mL. Both models were incubated for 24 h with RPMI 1640 medium (0.5 mL and 1.5 mL of the medium at the apical and basolateral sides, respectively), supplemented with 5% (v/v) FBS, penicillin (100 IU/mL), and streptomycin (100 mg/mL), 1% (v/v) sodium pyruvate, 25 µg/mL ECGS, and 25 µg/mL sodium heparin. After 24 h, the medium was discarded, and a complete fresh medium, further supplemented with 200 nM of Dex and 1% (v/v) ITS, was added to each insert. Cells were kept under liquid–liquid conditions (LLC) for 48 h and then transferred to air–liquid conditions (ALI), in which the medium from the apical side was removed and the medium from the basolateral side was replaced by 0.5 mL of fresh pre-heated complete medium (including Dex and ITS). Cells were kept under ALI until the day of the experiment (day 5).

Transepithelial electrical resistance (TEER) was measured every 2 days and also during the permeability assay to evaluate the integrity of the cell barrier. During the culturing of the models at LLC and throughout the permeability assay, TEER was measured directly by placing the electrode, connected to an EVOM^2^ Voltohmmeter (both from World Precision Instrument, Sarasota, USA), on the lateral side of the insert. However, during the ALI culture conditions, 0.5 mL and 1 mL of culture medium were added to each well, on the apical and basolateral sides, respectively, for 1 h at 37 °C with a humidified atmosphere of 5% CO_2_, before TEER measurements. This parameter was calculated based on Eq. 5:

5 $$\mathrm{Teer}=\left({\mathrm{TEER}}_{\mathrm{sample}}-{\mathrm{TEER}}_{\mathrm{blank}}\right)\times\mathrm{Insert}\;\mathrm{Area}$$

Insulin permeability was investigated as described elsewhere [22, 30]. 0.5 mL of insulin (control), mPEG-i-ZNPs, mPEG-F-i-ZNPs, P407-i-ZNPs, and P407-F-i-ZNPs, contained 50 µg/mL of insulin, diluted in HBSS buffer (pH 7.4) were added to the apical chamber, and the well plates were shaken at 100 rpm at 37 °C. Aliquots of 200 µL were withdrawn from the basolateral chamber at different time points (10, 30, 60, 90, 120, and 180 min) and replaced with the same volume of pre-warmed HBSS buffer (pH 7.4) to maintain a constant medium volume. The amount of insulin permeated across cell monolayers was determined by enzyme-linked immunosorbent assay (ELISA), which will be further described in the next section.

The cumulative permeability of insulin across cell monolayers was calculated from the concentrations measured in the basolateral compartment (Eq. 6), and the apparent permeability coefficients (*P*_app_) were calculated using Eq. 7:

6 $$\mathrm{Insulin}\;\mathrm{cumulative}\;\mathrm{permealibility}\,\left(\%\right)=\,\left(\frac{\mathrm{Total}\;\mathrm{insulin}\;\mathrm{mass}}{\mathrm{Theoritical}\;\mathrm{insulin\;mass}}\right)\times100$$

7 $${\mathrm P}_{\mathrm{app}}=\left(\frac{\triangle Q}\triangle\right)\times\left(\frac1{\mathrm A\times{\mathrm C}_0}\right)$$

where Δ*Q*/Δ*t* is the steady-state flux (µg/s), *C*_0_ is the initial insulin concentration in the apical compartment (µg/mL), and *A* is the membrane area of the insert (cm^2^). The *P*_app_ values were estimated using *C*_0_ values based on the complete insulin release from NPs. The experiments were carried out in triplicate (*n* = 3).

#### Enzyme-linked immunosorbent assay

Sandwich ELISA was used to quantitatively analyze permeability assay samples. Briefly, high-binding 96-well plates (Corning Costar, Durham, NC, USA) were coated with 50 µL of anti-insulin antibody (Abcam, ref. ab8304) diluted 1:1000 in PBS and incubated overnight at 4 °C under orbital agitation at 65 rpm. After incubation, wells were washed three times with 200 µL of PBST and blocked with 100 µL of 4% skimmed milk (PanReac AppliChem, Barcelona, Spain) diluted in PBST (0.05% Tween-20 in PBS) for 1 h at RT. Wells were washed again three times with 200 µL of PBST. An insulin calibration curve was prepared in the range of 10 to 2000 pg/mL. Fifty microliters of standards and diluted samples were added in triplicates to wells and incubated for 2 h at RT. Wells were washed as described before, and 50 µL per well of HRP anti-insulin antibody (Abcam, ref. ab28063), diluted 1:2000 in PBS, were added for 1 h at RT, followed by a washing step. Afterward, 100 µL per well of 1-Step™ Ultra TMB-ELISA Substrate Solution (Thermo Fisher Scientific, Rockford, IL, USA) were added and incubated in the dark for 15 min. Then, 100 µL of 2 M of sulfuric acid was added to each well to stop the reaction. Absorbance was measured at 450 nm using a microplate reader (SynergyTM Mx HM550, BioTek).

#### Statistical analysis

All experiments were performed at least in triplicates (*n* ≥ 3), and results are reported as mean or mean ± standard deviation (s.d.). Statistical significances were analyzed by one-way analysis of variance (ANOVA) for the quantification of hFcRn expression in human lung cell lines by flow cytometry followed by Dunnett’s multiple comparison test, and for the Papp followed by Sidak’s multiple comparisons test. Two-way ANOVA was used for the cumulative insulin release from ZNPs formulations followed by Dunnett’s multiple comparison test and for the in vitro cumulative permeability analysis followed by Dunnett’s multiple comparison test (GraphPad Prism, GraphPad Software Inc., CA, USA). The levels of significance were set at probabilities of *p < 0.05, **p < 0.01, ***p < 0.001, and ****p < 0.0001.

## Results and discussion

### Preparation and characterization of zein nanoparticles formulations

ZNPs were produced by the desolvation method, as described in the literature [18, 20], and insulin was used as a protein model to be loaded in ZNPs. The FcRn-targeted peptide was selected based on Mezo et al.’s work [23] and modified according to Sockolosky et al. and Datta-Mannan et al. [24, 25]. Theoretically, the added cysteine to the N-terminal of the peptide framework (**C**QRFVT**GHFGGLY**PANG), will not affect the binding affinity of the peptide to the FcRn, since the amino acids responsible for the binding to FcRn, **GHFGGLY**, were not change vat acidic pH was confirmed using surface plasmon resonance (data not shown).

**Fig. 2:**
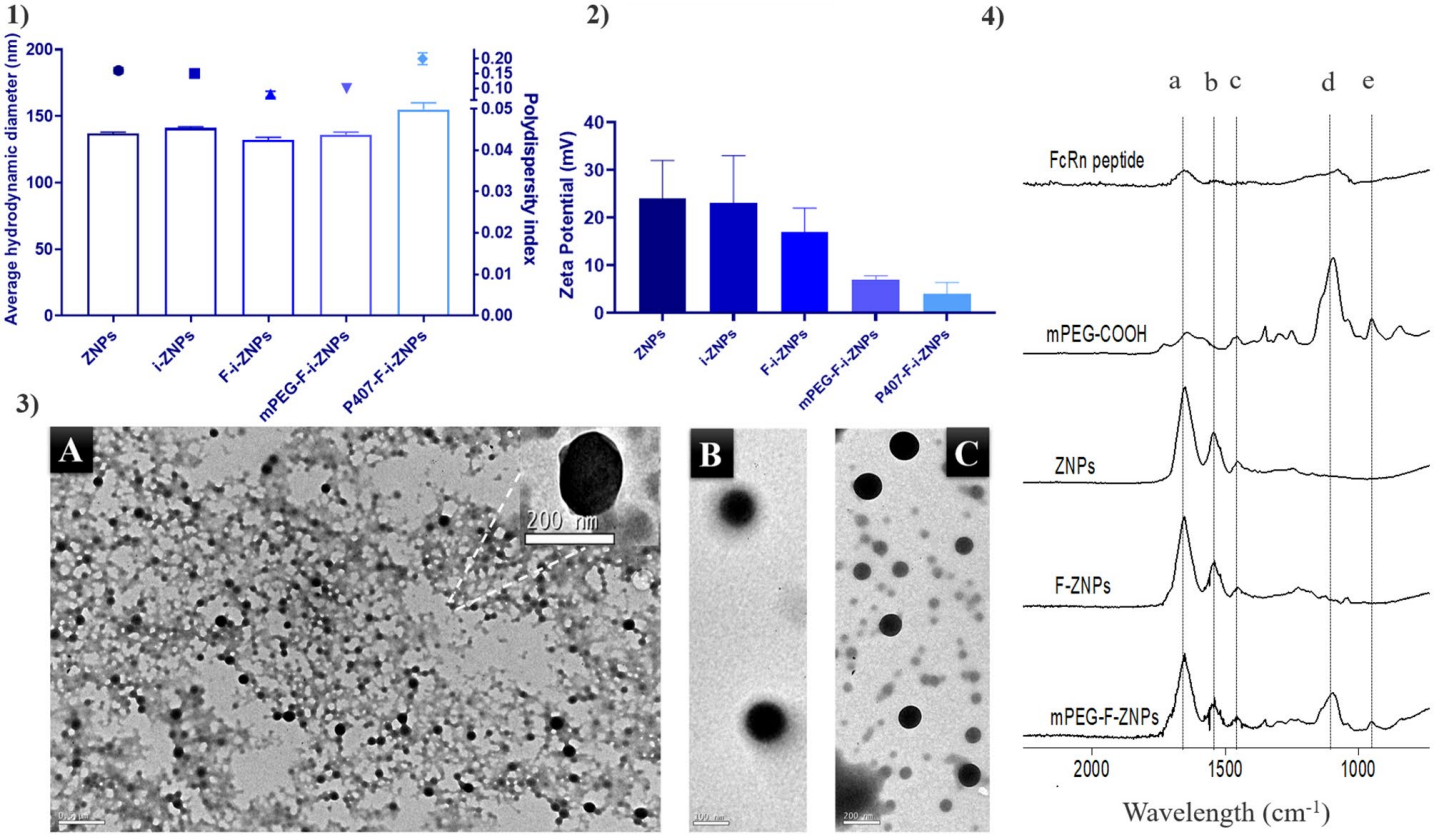
(**1**) The average hydrodynamic diameter and PdI measured by DLS. Results are expressed as mean ± s.d. (*n* = 3). (**2**) ZP were obtained using LDE. Results are expressed as mean ± s.d. (*n* = 3). (**3**) TEM images of (A) ZNPs, (B) targeted ZNPs covered with mPEG (mPEG-F-ZNPs), and (C) targeted ZNPs covered with P407 (P407-F-ZNPs) showing the physically adsorbed P407 on their surfaces. (**4**) FTIR spectra of FcRn-targeted peptide, mPEG-COOH, ZNPs, F-ZNPs, and mPEG-F-ZNPs. Dashed lines correspond to (a) 1647 cm^−1^ band belonging to the amide I group; (b) 1542 cm^−1^ band belonging to the amide II group; (c) 1455 cm^−1^ band belonging to the amide III group; (d–e) 1091 and 950 cm^−1^ bands belonging to the carboxylic group of mPEG

Empty and loaded ZNPs were successfully obtained by the desolvation technique without using a surfactant agent, demonstrating a mean hydrodynamic diameter of 137 and 141 nm, respectively, and a PdI lower than 0.2 (Fig. 2(1)). The obtained ZPs for both ZNPs showed a positive charge of 24 mV (Fig. 2(2)). This positive charge was reported in the literature for ZNPs produced without surfactant [20] and for pure zein charge [16]. The obtained ZNPs showed stability for up to 51 days at 4 °C in the dark. After that time, ZNPs started to aggregate as shown by their hydrodynamic diameters, whereas their ZNPs were similar to the first day of production (Table S1). Different studies reported that the stability of ZNPs varies according to the way that the formulation is stored, namely in dark or exposed to light, at cold or RT [31]. However, the reported works used surfactant agents to produce ZNPs. Nunes et al. produced ZNPs without surfactant agents by nanoprecipitation technique and reported stability of 1 month [20]. Nevertheless, the longer stability reported in this study might be explained by the production of ZNPs under acidic pH, which was lower than the isoelectric point of zein, 6.2 [31]. This might be stabilized by the repulsion effect of their positive charges (24 ± 8 mV). Loaded ZNPs were subjected to ultracentrifugation and analyzed the supernatants by micro-BCA assay to evaluate their insulin loading, which was around 7% (Table 1). The prepared calibration curve of insulin showed a linearity with *R*^2^ = 0.9934 (Fig. S1). The obtained insulin AE of i-ZNPs (approximately 72%) was lower than what was previously reported [18], but still, it has sufficient insulin loading to achieve high bioavailability. However, the DL and the AE of insulin of other formulations were estimated rather than analyzed because of possible interference of conjugated FcRn peptide, mPEG, and P407, using the ultracentrifugation method which is based on the molecular weight difference. FcRn peptide has a 1.8 kDa molecular weight which is lower than insulin of 5.8 kDa. In addition to the PEG molecular weight which is around 5 kDa, PEG-protein interactions are expected as well [32]. Therefore, the estimated quantity of insulin for F-i-ZNPs should be the same as i-ZNPs because it was subjected to acidic pH (5.3) environment during the FcRn conjugation reaction which is the same isoelectric point of insulin, and then insulin is not expected to be released, whereas the P407-F-i-ZNPs formulation is expected to have the same insulin DL and AE of F-i-ZNPs and i-ZNPs, since P407 is expected to stabilize the entire system of F-i-ZNPs. However, for the mPEG-F-i-ZNPs formulation estimation, it will be explained later in the “insulin release profile” section.

**Table 1:** Insulin loading and association efficiency of loaded ZNPs (i-ZNPs) as well as FcRn-targeted peptide conjugation efficiency of targeted unloaded ZNPs (F-ZNPs) were determined using the micro-BCA assay. Data are represented as mean ± s.d

	**Insulin DL (%)**	**Insulin AE (%)**	**FcRn-peptide CE (%)**
i-ZNPs	7 ± 1*	72 ± 7*	-
F-ZNPs	-	-	68 ± 8**

(*n = 3, **n = 6)

Targeted ZNPs were produced by covalent conjugation of the freshly prepared i-ZNPs to the FcRn-targeted peptide to form a stable, covalent amide bond, using EDC/NHS chemistry, which is considered safe and highly efficient [33]. The reaction was optimized in terms of buffer’s pH at different acidic pH closer to neutral pH and reaction time of different time points up to 12 h [34], preliminary data. However, the activation of the carboxylic acid of ZNPs worked in an acidic buffer (MES buffer, pH 5.3) for 1 h to avoid the release of insulin, which has an isoelectric point of 5.3.

The CE of the FcRn-targeted peptide was evaluated indirectly through the quantification of the filtrates collected during the washing steps and analyzed using micro-BCA assay. The prepared calibration curve of insulin showed a linearity with *R*^2^ = 0.9932 (Fig. S2), and the CE was around 68% (Table 1). However, the CE of the peptide was evaluated for the other formulations; mPEG-F-i-ZNPs and P407-F-i-ZNPs, because of possible collection of filtrates containing unconjugated FcRn peptide, nonadsorbed P407, or unconjugated mPEG. The hydrodynamic size and PdI of the targeted ZNPs (F-i-ZNPs) were not different from the non-targeted ZNPs (i-ZNPs), but the ZP decreased to 17 mV, due to the formation of the amide bonds and the peptide covering some of the surfaces of ZNPs, whereas the ZP was further dropped to 7 mV for the mPEG-F-i-ZNPs and 4 mV for the P407-F-i-ZNPs, which might further be explained by the covering of PEG to their surfaces and then changing the surrounded counterions of the NPs surfaces. However, measured ZPs can be related to the pH of the produced NPs systems which are different; the ZNPs and i-ZNPs produced in acidic pH of 5.3 and the other formulations might be shifted to an acidic pH range of 6–6.5 due to the carried carbodiimide reactions, as might be related to the results of Xu et al. [33].

Although zein is an amphiphilic protein and contains both functional groups (carboxylic acids and amines), ZNPs showed stability over the reaction process. It can be explained as the following; at pH 5.35, the carboxylic acids (pKa ~ 5) are deprotonated, while the amine groups (pKa ~ 10) are not reactive. Whereas when the reaction continues to form the amide bonds with the protonated active amine groups of the peptide which is already dissolved in pH 8.5, the amines from the peptide are faster to interact with the activated carboxylic acid of the ZNPs than the amines on the ZNPs. Immediately after removing the by-products, the reaction continues at pH 8.5 to covalently PEGylate the F-i-ZNPs, which is optimized to be conducted within 1 h. On the other hand, the physically adsorbed targeted ZNPs were processed at 4 °C overnight, without further optimization of the protocol. Covalently PEGylated ZNPs presented a similar mean hydrodynamic diameter, whereas the physically adsorbed ZNPs showed a slight increase in the hydrodynamic size, which was 155 nm, besides a trend of higher PdI of 0.2 (Fig. 2(1)). TEM images revealed a spherical shape but higher heterogenicity and lower sizes than the ones obtained by DLS (Fig. 2(3)). This might be due to the preparation method used, since TEM imaging involves samples drying before measurement, unlike DLS, in which samples were diluted in 10 mM sodium chloride solution (Fig. 2(3)) [35]. In addition, SEM images confirmed the spherical shape but showed aggregated forms of concentrated ZNPs (Fig. S3). However, the tested sample was subjected to the freeze-drying process, which may have caused morphological changes to ZNPs, but it can be optimized [20]. However, it is worth mentioning that this was not studied in this work.

In order to confirm the amide bond formation for both carbodiimide reactions, the FTIR spectra for F-ZNPs and mPEG-F-ZNPs, were compared with that of the individual compounds used in the preparation, namely FcRn-targeted peptide and mPEG-COOH. For the analysis of spectra, each spectrum was normalized and transferred to absorbance to detect weak signals beside very strong signals. In ZNPs as well as in targeted and PEGylated F-ZNPs, a band centered at about 1647 cm^−1^ attributed to the C = O stretching vibration of amide I was detected. In addition, the –N–H bending coupled to –C-N stretching vibration which represents amide II was observed at 1542 cm^−1^. In the spectra of mPEG-COOH and mPEG-F-ZNPs, the bands corresponding to the polymer fingerprint region, namely 1091 and 950 cm^−1^, were presented. However, due to the overlapping of bands corresponding to the amides’ bands, neither the covalent PEGylation nor the covalent FcRn-targeted peptide conjugation could show a formation of new amide bonds with sufficient resolution.

Modifying the surface of NPs with PEG is a common method to produce mucopenetration NPs, decrease the immunogenicity of bioactive drugs, including proteins and peptides, by avoiding the protein corona phenomena, and enhance solubility and stability. In addition, it has been used for both liquid and powder formulations of inhaled proteins, specifically PEG with small molecular weight (< 10 kDa) is often used as excipients in oral, intravenous, and nasal formulations [2]. In this context, the length of PEG chains and the surface density influence the properties of the PEG shell and its interaction with the mucus. It was demonstrated that NPs coated with long PEG chains (≥ 10 kDa) had more mucoadhesive properties than those coated with shorter PEG chains that showed mucosal diffusion properties [36]. In order to coat the ZNPs with PEG, covalent or non-covalent conjugation can be applied [2]. Physically adsorbed PEG (35 kDa) on ZNPs was described in the literature for oral insulin delivery [37], which might not enhance the mucodiffusive characteristics of the designed NPs, as discussed above. Moreover, this successful physically adsorbed PEGylation might be a result of the protein and long PEG chain strong interactions, which are described elsewhere [32]. On the other hand, covalent PEGylation of zein protein, before ZNPs production or developing zein micelles, works for encapsulating hydrophobic drugs [38, 39] which was not applicable in our study. Non-covalent PEGylation of approximately 5 kDa using poloxamer 407, a triblock copolymer of PEG and -polypropylene glycol (PPG), showed that mucus penetrating NPs performed better than non-modified NPs [40, 41]. In addition, poloxamer 407 is considered safe, low cost, and approved by medicine regulatory agencies including FDA and EMA [42]. Non-covalent PEGylation provides reversible dissociation of PEG chains from the particle surface over time due to the lack of a permanent covalent linkage [43]. Covalent PEGylation using mPEG, which is a biocompatible and non-biodegradable polymer, can be more suitable for bioconjugation of peptides and proteins since these monofunctional reactive polymers do not lead to the cross-linking of the poly-peptidic targets [44]. However, after crossing the mucus barrier, the functionalized NPs must interact with FcRn, and the PEG coating density might negatively affect the receptor recognition of the active targeting NPs with ligands anchored on the distal ends of the PEG. Nevertheless, the lower PEG surface density contributes to achieving the highest specificity and targeting efficiency [45, 46]. This is confirmed by the previous work of Han et al., where they showed the importance of considering the above-mentioned factors to achieve the recognition of the peptide by the receptor [47].

Stealth active targeting NPs strategy was the ligand buried under the PEGylated layer. Our methodology involved the coating process with PEG, after the functionalization of ZNPs. The concern regarding the possible masking of the FcRn-targeted ligand might be avoided by proper PEGylation density and PEG chain length, as discussed above [36, 40, 41, 47]. The reported work studied the hyaluronic acid-based NPs effect at the biological level, in terms of the effect of used PEG density on the blood circulation time, cellular uptake, and in vivo anticancer activity. Based on their results, they concluded that an appropriate degree of PEGylation is crucial to improve the targeting ability of ligand-buried PEGylated NPs [47]. Another evidence that supported the chosen strategy is that the ligand-buried PEGylated NPs may avoid protein corona formation, which could significantly hinder their specific ligand-receptor interactions, as well as enhance their elimination [48, 49].

Overall, the covalent PEGylation of ZNPs needs to be optimized and confirmed using other techniques, such as nuclear magnetic resonance (NMR) and X-ray photoelectron spectroscopy (XPS). It is worth mentioning that another strategy was used before to formulate the targeted PEGylated ZNPs using the maleimide of the PEG and the free thiol group of the cysteine on the N-terminal side of the peptide. However, no functionalization of the peptide was achieved (data not shown), which might be explained by strong PEG-protein interactions that can affect the availability of the maleimide ring and sterically hinder its binding to the cysteine of the peptide [32].

### Insulin release profile

**Fig. 3:**
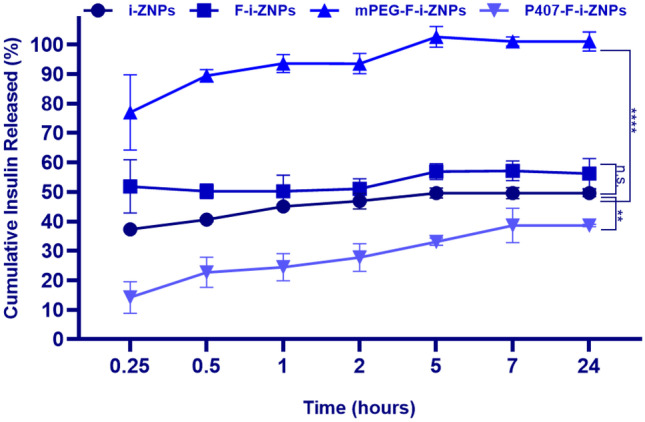
Cumulative insulin release from ZNPs at different steps of preparation: i-ZNPs, F-i-ZNPs, mPEG-F-i-ZNPs, and P407-F-i-ZNPs. The experiments were conducted for 24 h, at 37 °C in PBS buffer at pH 7.4. Results are expressed as mean ± s.d. (*n* = 3). Two-way ANOVA was used for statistical analysis (time and formulations, versus cumulative insulin released) followed by Dunnett’s multiple comparison test. The level of significant differences was set at probabilities of *p < 0.05, **p < 0.01, ***p < 0.001, and ****p < 0.0001

The release of insulin from the different ZNPs formulations during their incubation for 24 h in PBS at physiological pH is shown in Fig. 3. Both non-targeted and targeted ZNPs (i-ZNPs and F-i-ZNPs, respectively) displayed a similar insulin release profile. There is a burst release of insulin after 15 min of incubation (around 38% for i-ZNPs and 50% for F-i-ZNPs), similar to Nunes et al. [20], and then a stable release until 24 h, which might be explained by the adsorption of insulin on the surface of NPs. In order to estimate the insulin DL and AE of mPEG-F-i-ZNPs formulation, it relied on the F-i-ZNPs release assay results; where it has 50% release after 1 h which is the time required for the covalent PEGylation, so the DL and AE will be half of the quantified i-ZNPs. Therefore, the PEGylated ZNPs showed different patterns, namely the covalent PEGylated ZNPs (mPEG-F-i-ZNPs) showed a complete release (approximately 100%) after 5 h of incubation in PBS at pH 7.4, while P407-F-i-ZNPs showed a consistent and slower release pattern over time, with only 33% of insulin release after 5 h of the experiment. In fact, previously reported physically adsorbed PEGylated ZNPs using 35 kDa PEG and including lysine in the coating of ZNPs showed a slower and continuous release pattern, regardless of the media pH [18]. This provides evidence of the importance of using surfactant agents in NPs production. Therefore, the proposed formulation strategy in this study might not be proper for peptide drug delivery, and the method needs to be optimized. On the other hand, P407-F-i-ZNPs might be favorable to deliver insulin, since they showed a continuous and stable release pattern.

### FcRn expression in human lung cell lines

**Fig. 4:**
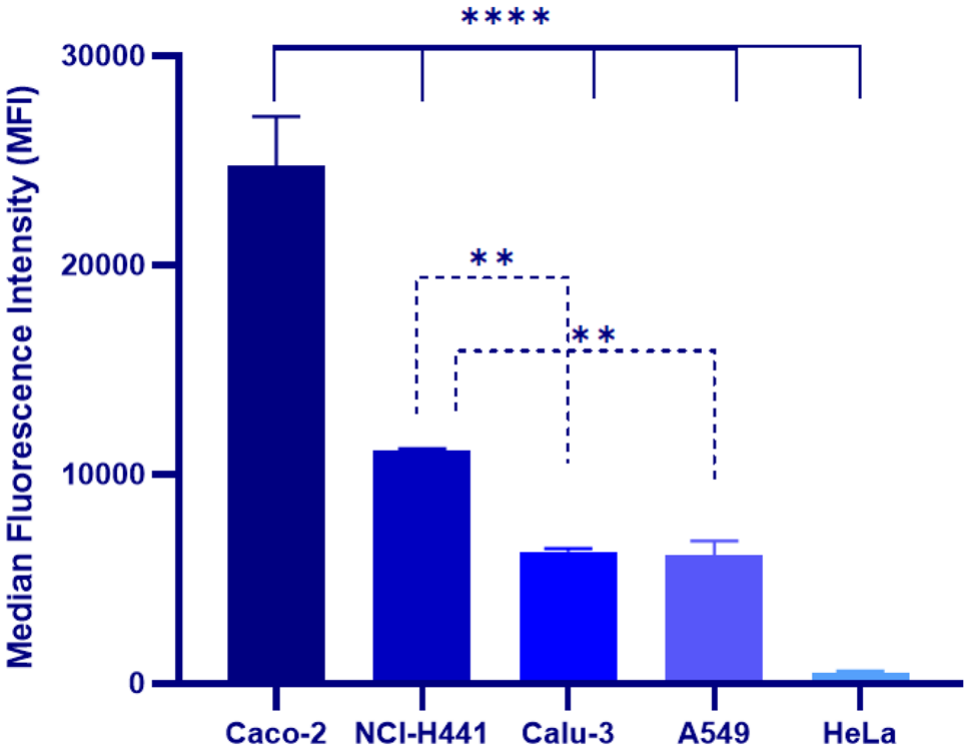
Quantification of hFcRn expression in human lung cell lines by flow cytometry. Results are presented as median fluorescence intensity (MFI) (a.u.) for the positive control (Caco-2), the negative control (HeLa), and the lung cell lines (A549, Calu-3, and NCI-H441), probed with anti-hFcRn antibody, followed by anti-mouse Alexa 594 antibody. Results were presented as mean ± s.d. (*n* = 3). One-way ANOVA followed by Dunnett’s multiple comparison test were used for the statistical analyses. The level of significant differences was set at probabilities of *p < 0.05, **p < 0.01, ***p < 0.001, and ****p < 0.0001

A flow cytometry analysis was performed to confirm the expression of human FcRn (hFcRn) in human lung epithelial cell lines (A549, NCI-H441, Calu-3), while Caco-2 and HeLa cells were used as positive and negative controls, respectively. As shown in Fig. 4, the positive control Caco-2 cells expressed the hFcRn receptor, which was in accordance with the data reported in the literature [50]. This cell line is extensively used to evaluate the cellular uptake of FcRn-targeted NPs coated with albumin, IgG, or other FcRn-targeted ligands intended to be used for oral delivery [51–53]. On the other hand, the negative control HeLa cell line did not express the hFcRn receptor, as expected [54].

In order to select the lung cell line to evaluate the in vitro permeability of insulin loaded to targeted ZNPs, the expression of hFcRn in lung cell lines was quantitatively evaluated through flow cytometry (Fig. 4). The results showed that all lung cell lines tested, namely NCI-H441, A549, and Calu-3 cells, expressed hFcRn, with an MFI of 11,137 ± 100, 6310 ± 150, and 6171 ± 670, respectively, showing a significantly higher expression for the NCI-H441 cell line. It is important to highlight that the hFcRn expression in both A549 and Calu-3 cell lines was already reported in the literature [55–57], while no data were previously reported regarding the hFcRn expression in the NCI-H441 cell line. In this sense, to the best of our knowledge, we demonstrated for the first time that NCI-H441 cells also expressed hFcRn, as well as the other two lung cell lines (A549 and Calu-3).

Therefore, an in vitro lung model developed by Costa et al. with a potential tool to assess the transport of NPs across the air-blood barrier in a healthy state and proinflammatory state was used in this study, but with some modifications. [22]. These lung in vitro model cells are cultured on inserts, which is widely used for permeable cell culture, and allow cells to uptake and secrete molecules on the apical and basolateral sides [58]. This model is composed of epithelial NCI-H441 cells and differentiated THP-1 macrophage-like cells cultured on the apical side of the insert and endothelial HPMEC-ST1.6R cells on the basolateral side [22]. This developed 3D in vitro model mimics the alveolar-capillary membrane in terms of morphology as well as physiological behavior. In fact, the exposure of the epithelial cells to the air is mimicking more closely the in vivo exposure, where it is relevant to aerosol deposition [22], in addition to other parameters that can be influenced in case of ALI, such as the cellular barrier, cell morphology, and gene expression properties [59, 60]. Therefore, to assess the lung permeability of insulin-loaded ZNPs formulations (i-ZNPs, F-i-ZNPs, mPEG-F-i-ZNPs, and P407-F-i-ZNPs) via FcRn, this in vitro lung model was reproduced but without THP-1 cells, as explained before.

### Cell viability assay

**Fig. 5:**
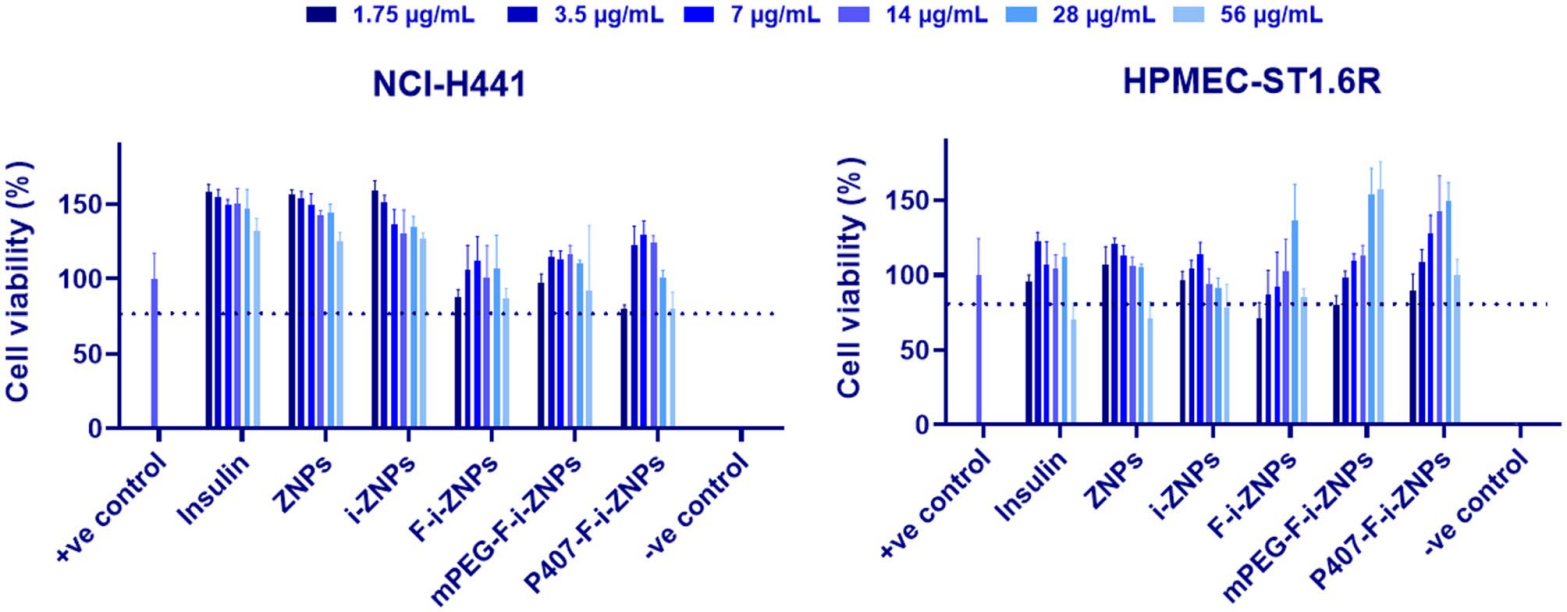
Cell viability of (**A**) NCI-H441 and (**B**) HPMEC-ST1.6R cell lines when exposed to insulin, empty ZNPs and insulin-loaded ZNPs (i-ZNP, F-i-ZNPs, mPEG-F-i-ZNPs, and P407-F-i-ZNPs), including positive control (+ve control) and negative control (-ve control) by resazurin assay. Data are presented as mean ± s.d. (*n* = 3)

The impact of free insulin, empty ZNPs, and insulin-loaded ZNPs (i-ZNP, F-i-ZNPs, mPEG-F-i-ZNPs, and P407-F-i-ZNPs) in the metabolic activity of the two cell lines that constitute the in vitro lung model of the air-blood barrier, namely NCI-H441 and HPMEC-ST1.6R cells (epithelial and endothelial cells, respectively), was studied by resazurin assay. Both cell lines were incubated with free insulin, empty, or insulin-loaded ZNPs for 24 h at 37 °C, in a range of insulin concentrations from 1.75 to 56 µg/mL. The results of the cellular viability of both cell lines are illustrated in Fig. 5. After 24 h of incubation of the cell lines with the treatments, the formulations showed around 70% cellular viability, which is considered the lower viability threshold, in the range of insulin concentrations [61].

### Insulin permeability in an in vitro lung model

In order to reach blood circulation, the targeted ZNPs developed in this study need to migrate from the lumen to the basal side of the pulmonary epithelium. Hence, after the flow cytometry validation of the FcRn expression on the in vitro cell culture model (namely NCI-H441 cells) and confirming the safety of the developed ZNPs on both cell lines of the lung model (NCI-H441 and HPMEC-ST1.6R), insulin permeability across the in vitro lung models was investigated, namely NCI-H441 monoculture model and NCI-H441 and HPMEC-ST1.6R co-culture model.

**Fig. 6:**
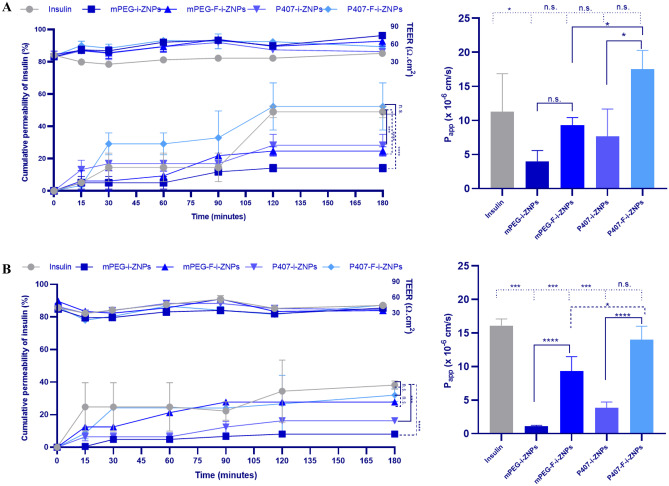
In vitro permeability studies. Cumulative permeability of insulin and apparent permeability coefficients (*P*_app_ × 10.^−6^ cm/s) across A NCI-H441 monoculture model and B NCI-H441 + HPMEC-ST1.6R co-culture model after incubation with insulin, targeted insulin-loaded ZNPs; mPEG-F-i-ZNPs, P407-F-i-ZNPs, and non-targeted insulin loaded ZNPs; mPEG-i-ZNPs and P407-i-ZNPs, for 180 min, contained 50 µg/mL of insulin, in HBSS at 37 °C. Results are represented as mean ± s.d. of three samples (*n* = 3). Two-way ANOVA was used for the statistical analysis of the cumulative permeability (time and formulations versus cumulative permeability of insulin) followed by Dunnett’s multiple comparison test, and one-way ANOVA was used for the *P*_app_ followed by Sidak’s multiple comparisons test. The level of significant differences was set at probabilities of *p < 0.05, **p < 0.01, ***p < 0.001, and ****p < 0.0001

After the addition of Dex and ITS, the confluent monolayer establishment and the development of tight junctions was assessed by monitoring the TEER from day 3 to day 5 of culture (Fig. S4). Results showed a non-statistically significant increase in TEER values until day 4 of culture and then started to stabilize and a non-statistically significant decrease as the cell differentiation is occurring (Fig. S4). Nonetheless, the TEER values for the in vitro lung monoculture model (NCI-H441 cells) and co-culture model (NCI-H441 and HPMEC-ST1.6R cells) were 74 ± 10 Ω.cm^2^ and 81 ± 14 Ω.cm^2^, respectively, obtained at the end of the culture period (5 days). This supports the successful development of continuous tight junctions between NCI-H441 cells [22, 62] and the integrity of the epithelial monolayer to mimic the in vivo scenario. For the last-mentioned reason, the TEER values were reported during the permeability experiment for each cumulative permeability result of each model, as represented in the upper section of the presented data on the cumulative permeability of insulin (Fig. 6).

The insulin cumulative permeability profiles from the NCI-H441 monoculture model incubated with free insulin, non-targeted insulin-loaded (mPEG-i-ZNPs and P407-i-ZNPs), and targeted insulin-loaded ZNPs (mPEG-F-i-ZNPs and P407-F-i-ZNPs) are presented in Fig. 6A. A non-significant difference in the amount of insulin permeating the pulmonary cells, immediately after 15 min of incubation for all the formulations including the control, slightly increases throughout the experiment (Fig. 6A). Compared with the non-targeted P407-i-ZNPs, after 180 min of the experiment, the permeability rate of insulin from the targeted P407-F-i-ZNPs was slightly higher but significant (Fig. 6A). Similarly in the co-culture model of NCI-H441 and HPMEC-ST1.6R cells, significantly higher cumulative permeability of insulin was obtained for the targeted P407-F-i-ZNPs compared to the non-targeted P407-i-ZNPs (Fig. 6B). Additionally, insulin permeability from P407-F-i-ZNPs was similar to free insulin, in both lung models. However, relying on the release assay results of P407-F-i-ZNPs (Fig. 3), which showed a controlled release of insulin within 24 h, it is expected to have a superior release of insulin over time.

Insulin *P*_app_ for the four formulations and the control (insulin) was calculated from the insulin permeability profiles across both lung models, after 180 min (Fig. 6). The *P*_app_ of P407-F-i-ZNPs recorded higher values, 17.5 × 10^−6^ cm s^−1^ and 13.8 × 10^−6^ cm s^−1^, than mPEG-F-i-ZNPs, 9.3 × 10^−6^ cm s^−1^ and 9.3 × 10^−6^ cm s^−1^, in monoculture and co-culture models, respectively. It is important to highlight that the insulin *P*_app_ for targeted insulin-loaded ZNPs (mPEG-F-i-ZNPs and P407-F-i-ZNPs) were significantly higher than non-targeted i-ZNPs, in the co-culture model of NCI-H441 and HPMEC-ST1.6R cells (Fig. 6B**)**. Overall, the addition of the FcRn-targeted ligand on the surface of ZNPs revealed to further increase insulin permeability across both lung models. Nevertheless, in accordance with the data from the cumulative permeability of insulin, the obtained results of the *P*_app_ of the targeted formulations did not exceed the *P*_app_ of the control, free insulin. That might be explained by a possible masking effect of the FcRn-targeted peptide that might hinder its ability to be recognized by the receptor. The critical factor to ensure the targeting efficiency is the ligand availability decorated on the surface of the targeted NPs [63]. However, the used strategy in this study was the ligand-buried strategy where a possible hindrance effect is possible [63], and therefore, a hindrance of the permeability across the in vitro model is expected. On the other hand, the existence of the targeting ligands on the NPs surfaces does not guarantee successful targeting, due to a possible buried-ligand phenomenon by adsorption of other molecules onto their surfaces in the biological environment or incorrect ligand orientation on their surfaces [63]. Therefore, further optimization of this formulation is needed, such as the degree of PEGylation [47], as well as confirming the covalent PEGylation, in order to improve the targeting ability by covalent linking the FcRn-targeted peptide to the distal part of the PEG. Nevertheless, the permeability of P407-F-i-ZNPs might be a result of the dissociation of the PEG chains from the particle surface over time, allowing the peptide to better interact with the receptor [43]. Therefore, the physically adsorbed P407-F-i-ZNPs formulation is promising to be used as a more stable nanocarrier for peptide delivery.

Both models showed the ability to permeate the formulations along with the control. However, the co-culture model better mimics the biological layers, because the formation of the thin thickness layer of the air-blood barrier through the existence of endothelial layer and Matrigel^®^ basement membrane is the preferred choice to be used over the monoculture model for the ZNPs permeability. However, in this study, an in vitro model which did not express mucus was used; therefore, it is recommended to conduct a further investigation using another appropriate model.

## Conclusions

The revolution of targeted nanomedicine is supporting the clinical landscape by providing new potent therapeutic strategies, besides introducing solutions to overcome the shortcomings of conventional therapies. Pulmonary delivery is favored over other non-invasive routes, due to the ability to bypass the classical oral delivery barriers that affect their absorption. Such an ideal nanocarrier system for pulmonary administration should overcome the barriers imposed by the pulmonary tract to increase drug permeation across the lung. In this work, we developed and characterized candidate protein delivery systems for the pulmonary administration of peptides. The developed ZNPs had a small size and narrow size distribution, which was revealed to be suitable for pulmonary translocation. The coating of the core insulin-loaded ZNPs with P407 showed insulin protection from premature release. The in vitro cell culture models consisting of NCI-H441 epithelial cell line and HPMEC-ST1.6R endothelial cell line were used herein. The FcRn expression in NCI-H441 cells was confirmed by flow cytometry analysis. Moreover, the safety of ZNPs was evaluated after incubating both cell lines, NCI-H441 and HPMEC-ST1.6R, for 24 h with the nanosystems. Targeted insulin-loaded ZNPs enhanced insulin permeation across both lung models (lung monoculture model (NCI-H441 cells) and co-culture model (NCI-H441 and HPMEC-ST1.6R cells)) than non-targeted ZNPs, except mPEG-F-i-ZNPs in the monoculture model. Ultimately, this study shows further possibilities for the development of FcRn-targeted therapies while fostering the discussion on enhancing pulmonary absorption of peptides intended to treat various pathologies. In addition, it opens the avenues for researchers to apply PEGylated ZNPs for pulmonary delivery. Overall, the targeted PEGylated ZNPs showed to be a suitable drug carrier, and the P407-F-i-ZNPs showed to adequately fit the demands of delivery systems aimed for pulmonary administration. In addition, the used FcRn-targeted ligand showed a role in potentiating the success of pulmonary ZNPs to cross the pulmonary epithelium, which is naturally limited. However, more studies using an in vitro model which secretes mucus are needed to mimic the lung environment. Despite the obtained characterization of the developed ZNPs and their capacity to increase insulin permeability across the in vitro cell culture models tested, in vivo studies will be essential to determine the success of this formulation, after its administration through the pulmonary route using a transgenic mouse model expressing hFcRn.

## Supplementary Information

Below is the link to the electronic supplementary material.

Supplementary file1 (DOCX 701 KB)

## Data Availability

The collected and analyzed datasets during this study are available from the corresponding author on reasonable request.

## References

[1] Muttenthaler M, King GF, Adams DJ, Alewood PF. Trends in peptide drug discovery. Nat Rev Drug Discov. 2021;204. Nature Publishing Group. 2021;20:309–25. 10.1038/s41573-020-00135-8. 10.1038/s41573-020-00135-8 33536635

[2] Matthews AA, Ee PLR, Ge R. Developing inhaled protein therapeutics for lung diseases. Mol Biomed. Springer. 2020;1:11. 10.1186/s43556-020-00014-z. 10.1186/s43556-020-00014-z PMC7595758 34765995

[3] Loira-Pastoriza C, Todoroff J, Vanbever R (2014). Delivery strategies for sustained drug release in the lungs. Adv Drug Deliv Rev Elsevier.

[4] Wang Y-B, Watts AB, Peters JI, Williams RO (2014). The impact of pulmonary diseases on the fate of inhaled medicines–a review. Int J Pharm Elsevier.

[5] Ganguly K, Carlander U, Garessus EDG, Fridén M, Eriksson UG, Tehler U (2019). Computational modeling of lung deposition of inhaled particles in chronic obstructive pulmonary disease (COPD) patients: identification of gaps in knowledge and data. Crit Rev Toxicol Taylor & Francis.

[6] Sultan MH, Mahdi WA, Kwon YM. Insulin release from NPH insulin-loaded Pluronic^®^ F127 hydrogel in the presence of simulated tissue enzyme activity. Process 2020, Vol 8, Page 1320. Multidisciplinary Digital Publishing Institute. 2020;8:1320. 10.3390/PR8101320.

[7] Goldberg T, Wong E. Afrezza (Insulin Human) Inhalation powder: a new inhaled insulin for the management of type-1 or type-2 diabetes mellitus. P T. MediMedia, USA. 2015;40:735–41. PMC4634344 26609206

[8] Hornby PJ, Cooper PR, Kliwinski C, Ragwan E, Mabus JR, Harman B, et al. Human and non-human primate intestinal FcRn expression and immunoglobulin G transcytosis. Pharm Res. Springer New York LLC. 2014;31:908–22. 10.1007/s11095-013-1212-3. 10.1007/s11095-013-1212-3 PMC3953555 24072267

[9] Kuo TT, Baker K, Yoshida M, Qiao S-W, Aveson VG, Lencer WI (2010). Neonatal Fc receptor: from immunity to therapeutics. J Clin Immunol Springer.

[10] Fan YY, Farrokhi V, Caiazzo T, Wang M, O’Hara DM, Neubert H. Human FcRn tissue expression profile and half-life in PBMCs. Biomol. 2019;9:373. Multidisciplinary Digital Publishing Institute. 2019;9:373. 10.3390/BIOM9080373. 10.3390/biom9080373 PMC6722552 31443181

[11] Low SC, Nunes SL, Bitonti AJ, Dumont JA (2005). Oral and pulmonary delivery of FSH–Fc fusion proteins via neonatal Fc receptor-mediated transcytosis. Hum Reprod Oxford Academic.

[12] Liang W, Pan HW, Vllasaliu D, Lam JKW. Pulmonary delivery of biological drugs. Pharm 2020;12:1025. Multidisciplinary Digital Publishing Institute. 2020;12:1025. 10.3390/PHARMACEUTICS12111025. 10.3390/pharmaceutics12111025 PMC7693150 33114726

[13] Guilleminault L, Azzopardi N, Arnoult C, Sobilo J, Hervé V, Montharu J (2014). Fate of inhaled monoclonal antibodies after the deposition of aerosolized particles in the respiratory system. J Control Release Elsevier.

[14] Azevedo C, Pinto S, Benjakul S, Nilsen J, Santos HA, Traverso G, et al. Prevention of diabetes-associated fibrosis: strategies in FcRn-targeted nanosystems for oral drug delivery. Adv Drug Deliv Rev. Elsevier. 2021;175:113778. 10.1016/j.addr.2021.04.016. 10.1016/j.addr.2021.04.016 33887405

[15] Depreter F, Pilcer G, Amighi K (2013). Inhaled proteins: challenges and perspectives. Int J Pharm Elsevier.

[16] Abdelsalam AM, Somaida A, Ayoub AM, Alsharif FM, Preis E, Wojcik M, et al. Surface-tailored zein nanoparticles: strategies and applications. Pharmaceutics. Multidisciplinary Digital Publishing Institute. 2021;1354. 10.3390/pharmaceutics13091354 10.3390/pharmaceutics13091354 PMC8465254 34575430

[17] Labib G. Overview on zein protein: a promising pharmaceutical excipient in drug delivery systems and tissue engineering. 10.1080/1742524720171349752. Taylor & Francis. 2017;15:65–75. 10.1080/17425247.2017.1349752. 10.1080/17425247.2017.1349752 28662354

[18] Reboredo C, González-Navarro CJ, Martínez-López AL, Martínez-Ohárriz C, Sarmento B, Irache JM. Zein-based nanoparticles as oral carriers for insulin delivery. Pharmaceutics. Multidisciplinary Digital Publishing Institute. 2021;14:39. 10.3390/pharmaceutics14010039. 10.3390/pharmaceutics14010039 PMC8779360 35056935

[19] Heep G, Almeida A, Marcano R, Vieira D, Mainardes RM, Khalil NM (2019). Zein-casein-lysine multicomposite nanoparticles are effective in modulate the intestinal permeability of ferulic acid. Int J Biol Macromol Elsevier.

[20] Nunes R, Baião A, Monteiro D, das Neves J, Sarmento B. Zein nanoparticles as low-cost, safe, and effective carriers to improve the oral bioavailability of resveratrol. Drug Deliv Transl Res. Springer. 2020;10:826–37. 10.1007/s13346-020-00738-z. 10.1007/s13346-020-00738-z 32207071

[21] Inchaurraga L, Martínez-López AL, Martin-Arbella N, Irache JM (2020). Zein-based nanoparticles for the oral delivery of insulin. Drug Deliv Transl Res.

[22] Costa A, de Souza C-W, Seabra V, Sarmento B, Lehr C-M (2019). Triple co-culture of human alveolar epithelium, endothelium and macrophages for studying the interaction of nanocarriers with the air-blood barrier. Acta Biomater Elsevier.

[23] Mezo AR, McDonnell KA, Castro A, Fraley C (2008). Structure-activity relationships of a peptide inhibitor of the human FcRn:human IgG interaction. Bioorganic Med Chem Bioorg Med Chem.

[24] Sockolosky JT, Tiffany MR, Szoka FC. Engineering neonatal Fc receptor-mediated recycling and transcytosis in recombinant proteins by short terminal peptide extensions. Proc Natl Acad Sci USA. National Academy of Sciences. 2012;109:16095–100. 10.1073/pnas.1208857109. 10.1073/pnas.1208857109 PMC3479544 22991460

[25] Datta-Mannan A, Boyles J, Huang L, Jin ZY, Peariso A, Murphy AT, et al. Engineered FcRn binding fusion peptides significantly enhance the half-life of a fab domain in cynomolgus monkeys. Biotechnol J. John Wiley & Sons, Ltd; 2019;14:1800007. 10.1002/biot.201800007. 10.1002/biot.201800007 29802766

[26] Schoubben A, Blasi P, Giovagnoli S, Perioli L, Rossi C, Ricci M (2009). Novel composite microparticles for protein stabilization and delivery. Eur J Pharm Sci Elsevier.

[27] Fernández-Urrusuno R, Calvo P, Remuñán-López C, Vila-Jato JL, Alonso MJ (1999). Enhancement of nasal absorption of insulin using chitosan nanoparticles. Pharm Res Springer.

[28] Ho D-K, Costa A, De Rossi C, de Souza C-W, Loretz B, Lehr C-M (2018). Polysaccharide submicrocarrier for improved pulmonary delivery of poorly soluble anti-infective ciprofloxacin: preparation, characterization, and influence of size on cellular uptake. Mol Pharm Pergamon.

[29] Kalashnyk O, Petrova Y, Lykhmus O, Mikhalovska L, Mikhalovsky S, Zhukova A (2013). Expression, function and cooperating partners of protease-activated receptor type 3 in vascular endothelial cells and B lymphocytes studied with specific monoclonal antibody. Mol Immunol Pergamon.

[30] Liu D, Bimbo LM, Mäkilä E, Villanova F, Kaasalainen M, Herranz-Blanco B (2013). Co-delivery of a hydrophobic small molecule and a hydrophilic peptide by porous silicon nanoparticles. J Control Release Elsevier.

[31] Pascoli M, de Lima R, Fraceto LF. Zein nanoparticles and strategies to improve colloidal stability: a mini-review. Front Chem. Frontiers Media S. A. 2018;6:6. 10.3389/fchem.2018.00006. 10.3389/fchem.2018.00006 PMC5810256 29473032

[32] Wu J, Zhao C, Lin W, Hu R, Wang Q, Chen H, et al. Binding characteristics between polyethylene glycol (PEG) and proteins in aqueous solution. J Mater Chem B. The Royal Society of Chemistry. 2014;2:2983–92. 10.1039/C4TB00253A. 10.1039/c4tb00253a 32261674

[33] Xu Y, Wei Z, Xue C, Huang Q (2022). Assembly of zein–polyphenol conjugates via carbodiimide method: evaluation of physicochemical and functional properties. LWT.

[34] Soe ZC, Ou W, Gautam M, Poudel K, Kim BK, Pham LM, et al. Development of folate-functionalized PEGylated zein nanoparticles for ligand-directed delivery of paclitaxel. Pharm 2019;11:562. Multidisciplinary Digital Publishing Institute. 2019;11:562. 10.3390/PHARMACEUTICS11110562. 10.3390/pharmaceutics11110562 PMC6920870 31671569

[35] Meewan J, Somani S, Laskar P, Irving C, Mullin M, Woods S, et al. Limited impact of the protein corona on the cellular uptake of PEGylated zein micelles by melanoma cancer cells. Pharmaceutics. MDPI. 2022;14. 10.3390/PHARMACEUTICS14020439/S1. 10.3390/pharmaceutics14020439 PMC8877401 35214171

[36] Tang W, Zhang Y, Zhu G (2022). Pulmonary delivery of mucosal nanovaccines. Nanoscale The Royal Society of Chemistry.

[37] Reboredo C, González-Navarro CJ, Martínez-Oharriz C, Martínez-López AL, Irache JM. Preparation and evaluation of PEG-coated zein nanoparticles for oral drug delivery purposes. Int J Pharm. Elsevier; 2021;597:120287. 10.1016/j.ijpharm.2021.120287. 10.1016/j.ijpharm.2021.120287 33524523

[38] Podaralla S, Averineni R, Alqahtani M, Perumal O (2012). Synthesis of novel biodegradable methoxy poly(ethylene glycol)-zein micelles for effective delivery of curcumin. Mol Pharm American Chemical Society.

[39] Song R, Zhou Y, Li Y, Yang Z, Li F, Huang Q, et al. Preparation and characterization of mPEG-g-α-zein biohybrid micelles as a nano-carrier. J Appl Polym Sci. John Wiley & Sons, Ltd; 2015;132. 10.1002/APP.42555

[40] Nunes R, Araújo F, Tavares J, Sarmento B, das Neves J. Surface modification with polyethylene glycol enhances colorectal distribution and retention of nanoparticles. Eur J Pharm Biopharm. Elsevier; 2018;130:200–6. 10.1016/j.ejpb.2018.06.029. 10.1016/j.ejpb.2018.06.029 29960016

[41] Melo M, Nunes R, Sarmento B, Das NJ (2019). Colorectal distribution and retention of polymeric nanoparticles following incorporation into a thermosensitive enema. Biomater Sci Royal Society of Chemistry.

[42] Bodratti AM, Alexandridis P. Formulation of poloxamers for drug delivery. J Funct Biomater 2018, Vol 9, Page 11. Multidisciplinary Digital Publishing Institute; 2018;9:11. 10.3390/JFB9010011. 10.3390/jfb9010011 PMC5872097 29346330

[43] Huckaby JT, Lai SK (2018). PEGylation for enhancing nanoparticle diffusion in mucus. Adv Drug Deliv Rev Elsevier.

[44] Vijay Kumar Thakur MKT. Handbook of Polymers for Pharmaceutical Technologies [Internet]. Thakur VK, Thakur MK, editors. Hoboken, NJ, USA: John Wiley & Sons, Inc.; 2015. 10.1002/9781119041375.

[45] Cruz LJ, Tacken PJ, Fokkink R, Figdor CG (2011). The influence of PEG chain length and targeting moiety on antibody-mediated delivery of nanoparticle vaccines to human dendritic cells. Biomaterials Elsevier.

[46] Hak S, Helgesen E, Hektoen HH, Huuse EM, Jarzyna PA, Mulder WJM (2012). The effect of nanoparticle polyethylene glycol surface density on ligand-directed tumor targeting studied in vivo by dual modality imaging. ACS Nano American Chemical Society.

[47] Han X, Li Z, Sun J, Luo C, Li L, Liu Y (2015). Stealth CD44-targeted hyaluronic acid supramolecular nanoassemblies for doxorubicin delivery: probing the effect of uncovalent pegylation degree on cellular uptake and blood long circulation. J Control Release Elsevier.

[48] Dai Q, Walkey C, Chan WCW. Polyethylene glycol backfilling mitigates the negative impact of the protein corona on nanoparticle cell targeting. Angew Chemie Int Ed. Angew Chem Int Ed Engl. 2014;53:5093–6. 10.1002/anie.201309464. 10.1002/anie.201309464 24700480

[49] Narum SM, Le T, Le DP, Lee JC, Donahue ND, Yang W, et al. Passive targeting in nanomedicine: fundamental concepts, body interactions, and clinical potential. Nanoparticles Biomed Appl Elsevier. 2020;37–53. 10.1016/B978-0-12-816662-8.00004-7.

[50] Sato K, Nagai J, Mitsui N, Ryoko Yumoto, Takano M. Effects of endocytosis inhibitors on internalization of human IgG by Caco-2 human intestinal epithelial cells. Life Sci. Pergamon. 2009;85:800–7. 10.1016/j.lfs.2009.10.012. 10.1016/j.lfs.2009.10.012 19879882

[51] Martins JP, Liu D, Fontana F, Ferreira MPA, Correia A, Valentino S, et al. Microfluidic nanoassembly of bioengineered chitosan-modified FcRn-targeted porous silicon nanoparticles @ hypromellose acetate succinate for oral delivery of antidiabetic peptides. ACS Appl Mater Interfaces. Am Chem Soc. 2018;10:44354–67. 10.1021/acsami.8b20821. 10.1021/acsami.8b20821 30525379

[52] Martins JP, D’Auria R, Liu D, Fontana F, Ferreira MPA, Correia A, et al. Engineered multifunctional albumin-decorated porous silicon nanoparticles for FcRn translocation of insulin. Small. John Wiley & Sons, Ltd. 2018;14:1800462. 10.1002/SMLL.201800462. 10.1002/smll.201800462 29855134

[53] Martins JP, Figueiredo P, Wang S, Espo E, Celi E, Martins B (2022). Neonatal Fc receptor-targeted lignin-encapsulated porous silicon nanoparticles for enhanced cellular interactions and insulin permeation across the intestinal epithelium. Bioact Mater Elsevier.

[54] Ishii-Watabe A, Saito Y, Suzuki T, Tada M, Ukaji M, Maekawa K (2010). Genetic polymorphisms of FCGRT encoding FcRn in a Japanese population and their functional analysis. Drug Metab Pharmacokinet Elsevier.

[55] Ferguson DC, Blanco JG. Regulation of the human Fc-neonatal receptor alpha-chain gene FCGRT by MicroRNA-3181. Pharm Res. Springer New York LLC. 2018;35:15. 10.1007/s11095-017-2294-0. 10.1007/s11095-017-2294-0 PMC5757240 29302759

[56] Vllasaliu D, Alexander C, Garnett M, Eaton M, Stolnik S (2012). Fc-mediated transport of nanoparticles across airway epithelial cell layers. J Control Release Elsevier.

[57] Bequignon E, Dhommée C, Angely C, Thomas L, Bottier M, Escudier E, et al. FcRn-dependent transcytosis of monoclonal antibody in human nasal epithelial cells in vitro: a prerequisite for a new delivery route for therapy? Int J Mol Sci. 2019;20:1379. Multidisciplinary Digital Publishing Institute. 2019;20:1379. 10.3390/IJMS20061379. 10.3390/ijms20061379 PMC6470570 30893823

[58] Sousa F, Castro P. Cell-based in vitro models for nasal permeability studies. In: Sarmento B, editor. Concepts model drug permeability stud cell tissue based Vitr Cult Model. Sawston: Woodhead Publishing. 2016;83–100. 10.1016/B978-0-08-100094-6.00006-7.

[59] Dvorak A, Tilley AE, Shaykhiev R, Wang R, Crystal RG (2011). Do airway epithelium air–liquid cultures represent the in vivo airway epithelium transcriptome? Am J Respir Cell Mol Biol. American Thoracic Society.

[60] Kreft ME, Jerman UD, Lasič E, Lanišnik Rižner T, Hevir-Kene N, Peternel L (2015). The characterization of the human nasal epithelial cell line RPMI 2650 under different culture conditions and their optimization for an appropriate in vitro nasal model. Pharm Res Springer.

[61] ISO 10993–5:2009 - Biological evaluation of medical devices — Part 5: Tests for in vitro cytotoxicity. 2009;34.

[62] Ren H, Birch NP, Suresh V. An optimised human cell culture model for alveolar epithelial transport. PLoS One. Pub Lib Sci. 2016;11:e0165225. 10.1371/JOURNAL.PONE.0165225. 10.1371/journal.pone.0165225 PMC5079558 27780255

[63] Dai Q, Bertleff‐Zieschang N, Braunger JA, Björnmalm M, Cortez‐Jugo C, Caruso F. Particle targeting in complex biological media. Adv Healthc Mater. John Wiley & Sons, Ltd. 2018;7:1700575. 10.1002/adhm.201700575. 10.1002/adhm.201700575 28809092

