# Supplementary material for: Functionalized zein nanoparticles targeting neonatal Fc receptor to enhance lung absorption of peptides
Source: Drug Deliv Transl Res. 2023 Jan 1;13(6):1699–715. doi: 10.1007/s13346-022-01286-4 (PMC10126044; doi:10.1007/s13346-022-01286-4)
Supplement: Supplementary file 1 — Supplementary file1 (DOCX 701 KB) [file 13346_2022_1286_MOESM1_ESM.docx]

**Supplementary Material**

**Table S1** Stability of empty ZNPs at 1, 7, 34 and 51 days after production by desolvation method and stored at 4 °C. ZNPs were characterized regarding hydrodynamic diameter, PdI, and ZP. Data shown as mean ± standard deviation.

| Day number | DLS analysis model | Hydrodynamic diameter (nm) | PdI | ZP (mV) |
| --- | --- | --- | --- | --- |
| 1 | Cumulant | 136.1 ± 0.5 | 0.106 ± 0.0 | 27.8 ± 1.0 |
| 7 | Cumulant | 137.3 ± 0.7 | 0.106 ± 0.0 | 27.1 ± 0.2 |
| 34 | Cumulant | 140.6 ± 3.7 | 0.119 ± 0.0 | 28.0 ± 0.9 |
| 51 | Contin | 164.0 ± 68.1 and 4436.0 ± 906.0 | - | 26.6 ± 2.5 |


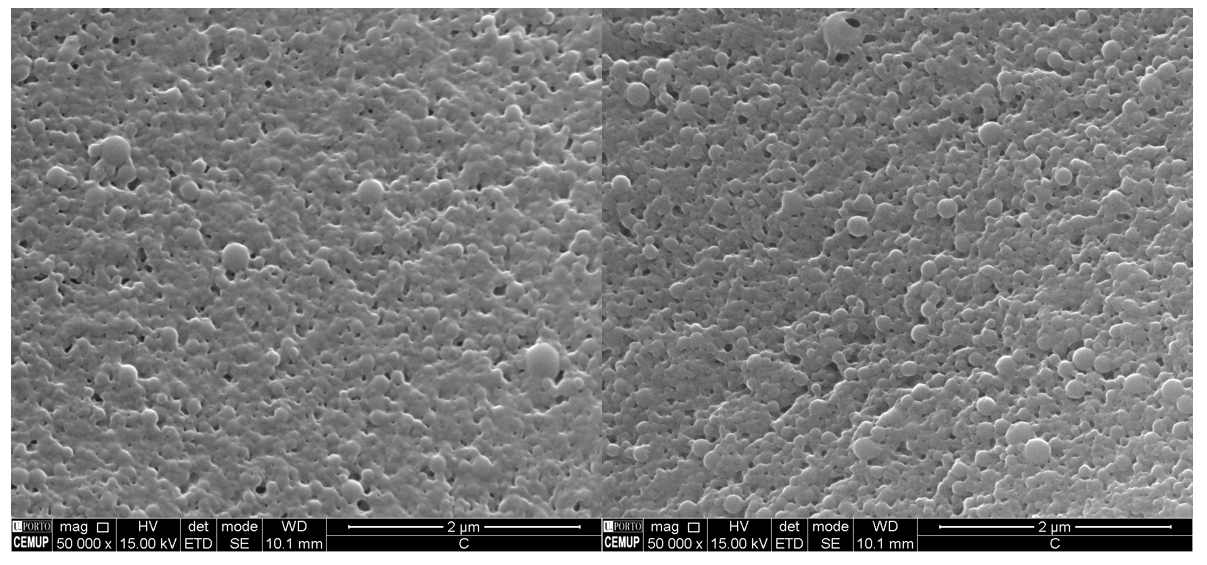


**Fig. S1** Representative SEM images of freeze-dried empty ZNPs prepared by desolvation method. Scale bars: = 2 μm


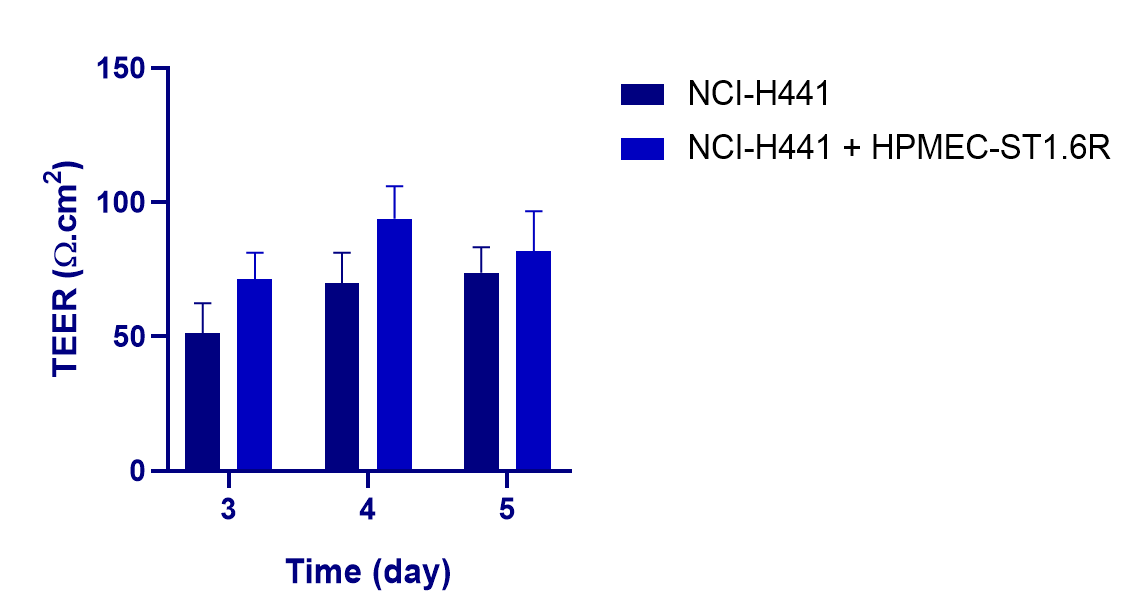


**Fig. S2** The TEER values of both NCI-H441 monoculture and NCI-H441 and HPMEC-ST1.6R co-culture models were assessed between days 3 and 5 of culture. Results are represented as mean ± s.d.(𝑛 ≥ 3)
